# A Control and Detecting System of Micro-Near-Infrared Spectrometer Based on a MOEMS Scanning Grating Mirror

**DOI:** 10.3390/mi9040152

**Published:** 2018-03-27

**Authors:** Haitao Liu, Zhiyu Wen, Dongling Li, Jian Huang, Ying Zhou, Pengfei Guo

**Affiliations:** National Key Discipline Laboratory of Novel Micro/Nano Devices and Systems Technologies of Chongqing University, Chongqing 400044, China; wzy@cqu.edu.cn (Z.W.); lidongling@cqu.edu.cn (D.L.); 20120801007@cqu.edu.cn (J.H.); yzhou@cqu.edu.cn (Y.Z.); 20150802004t@cqu.edu.cn (P.G.)

**Keywords:** micro-NIR spectrometer, scanning grating mirror, control, detecting, circuits

## Abstract

Based on the scanning grating mirror we developed, this paper presents a method of the precise control of a scanning grating mirror and of high-speed spectrum data detection. In addition, the system circuit of the scanning grating mirror control and spectrum signal detecting is designed and manufactured in this paper. The mirror control system includes a drive generator module, an amplitude detection module, a feedback control module, and a variable gain amplification (VGA) module; the detecting system includes a field programmable gate array (FPGA) main control module, a synchronous trigger module, an analog-digital conversion (ADC) module, and a universal serial bus (USB) interface module. The final results of the experiment show that the control system has successfully realized the precision control of the swing of the scanning grating mirror and that the detecting system has successfully realized the high-speed acquisition and transmission of the spectral signal and the angle signals. The spectrum has been reconstructed according to the mathematical relationship between the wavelength λ and the angle β of the mirror. The resolution of the spectrometer reaches 10 nm in the wavelength range of 800–1800 nm, the signal-to-noise ratio (SNR) of the spectrometer is 4562 at full scale, the spectrum data drift is 0.9% in 24 h, and the precision of the closed loop control is 0.06%.

## 1. Introduction

Micro-near-infrared (Micro-NIR) spectrometers have been widely used in food safety, environmental monitoring, industrial and agricultural production, aerospace and national security, and other fields in the last few decades due to its unique advantages of qualitative and quantitative detection of solid, liquid, and gas composition and content, It is non-contact, there is no need for sample pretreatment, it is fast, and there is high throughput [1,2,3,4].

At present, many commercialized micro-NIR spectrometer products have been introduced to the market. Such as Ocean Optics (Largo, FL, USA), Avantes (Apeldoorn, The Netherlands), and Hamamatsu Photonics (Tokyo, Japan), and so on. However, most of these products have a common characteristic of working with a fixed diffraction grating and an InGaAs detector array, and this configuration inevitably results in a production cost and large volume [5,6]. The appearance of the Micro Opto Electro Mechanical Systems (MOEMS) scanning grating mirror greatly changes the situation because the MOEMS scanning grating mirror can simultaneous diffract and scan the spectrum simultaneously. By replacing the traditional fixed diffraction grating with a MOEMS scanning grating mirror in a micro-NIR spectrometer, the cheap InGaAs single photodetector diode instead of the expensive InGaAs detector array can be used to detect the continuous spectrum [7,8,9,10].

All of the actuation mechanisms of the scanning grating mirror can be mainly divided into four categories: electromagnetic [11], electrostatic [12,13,14], electrothermal [15,16,17], and piezoelectric [18,19,20,21]. The electromagnetic actuation mechanisms have a relatively large size due to the external magnetic field; the mechanical properties are linear and the electromagnetic force is sufficiently large which leads to a large scanning angle [22]. The scanning angle is crucial to the detected spectral range for micro-NIR spectrometers; the 1% deviation of the scanning angle may cause approximately dozens of nanometer deviations of the detecting wavelength. Therefore, the scanning grating mirror must be accurately controlled.

At present, Germany’s Hiper Scan Company (Dresden, Germany) [23], Germany’s Fraunhofer Institute for Photonic Microsystems (IPMS) (Dresden, Germany) [8], and China’s Nanjing Intellisense Company(Jiangsu, China) [24] have developed their NIR spectrometers based on the scanning grating mirror in all of their systems. Because scanning gratings do not integrated a deflection angle sensor, the photodiode is used to replace the position-sensitive detector (PSD) to acquire the information about the deflection angle of the scanning mirror and the closed-loop control of the scanning mirror, which would enlarge and complicate the entire system. Furthermore, the data acquisition system is bulky and complex, which cannot meet the special application requirements of micro-NIR spectrometer based on the MOEMS scanning grating mirror.

Based on the scanning grating mirror that we developed, we here present a method of the precise control of this scanning grating mirror and of high-speed spectrum data detection. In addition, the system circuit of the scanning grating mirror control and spectrum signal detecting is designed and manufactured in this paper. The final results of the experiment show that the designed system can successfully achieve the precise control of the scanning grating mirror and obtain the spectrum data.

## 2. The Principle of the System Structure

**Figure 1:**
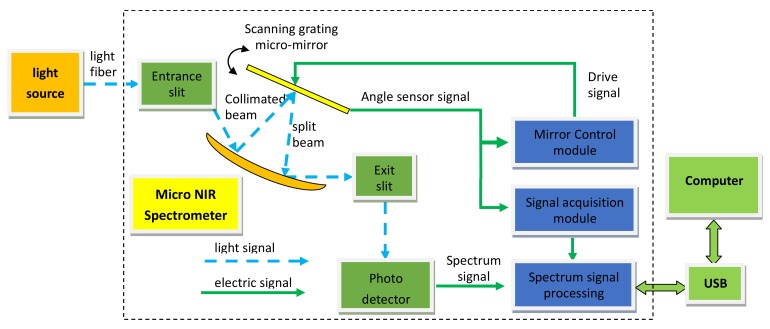
The system structure principle of a micro-NIR spectrometer.

Figure 1 shows the principle of the system structure of the micro-NIR spectrometer based on the MOEMS scanning grating mirror [25]. The light beam emitted from the NIR light source passes through the entrance slit to the concave mirror and irradiates on the surface of the MOEMS scanning grating mirror after it is collimated. Then, the monochromatic light is reflected through the concave mirror to the exit slit and detected by the InGaAs single photodetector diode. At the same time, the output angle signal of the integrated angle sensor on the surface of the scanning grating mirror acts as a reference signal for the closed-loop feedback control, and acts as a synchronous trigger signal for spectrum detection. Finally, the output of the InGaAs single photodetector diode and the output of the angle sensor signal are both transmitted to the computer through the universal serial bus (USB) interface for spectrum reconstruction, display and storage.

**Figure 2:**
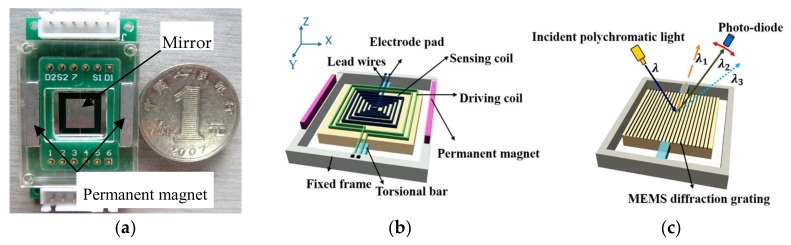
The Micro Opto Electro Mechanical Systems (MOEMS) scanning grating mirror fixed on the Printed Circuit Board (PCB) frame: (**a**) the fabricated scanning grating mirror, (**b**) the driving and angle sensing coils on the lower side and (**c**) the integrated blazed grating on the upper side.

This scanning grating mirror includes a movable mirror plate, a pair of torsional bars, integrated blazed grating, driving and angle sensing coils, lead wires, electrode pads, and a pair of permanent magnets [22]. Figure 2 shows the schematic diagram of a MOEMS scanning grating mirror. The movable mirror plate is attached to the outer fixed frame via a pair of rectangular torsional bars. The length and width of the movable mirror is 6 mm × 6 mm, and the thickness is 0.5 mm. One side of this device integrates the blazed grating, the density of the grating lines is 250 line/mm, the coating material on the surface is Al; and the other integrates the driving and angle sensing coils, the coating material on the surface is Au. The driving and angle sensing coils are connected to the electrode pads by lead wires that cross the torsional bars. Besides this, a pair of permanent magnets are assembled and generate a magnetic field, therefore, so the Lorentz force actuates the mirror plate scanning around the beam. The induced electromotive force is generated through the angle sensing coil due to the reciprocating motion of movable mirror plate in the magnetic fields. Meanwhile, the integrated blazed grating can simultaneously diffract and scan the spectrum.

**Figure 3:**
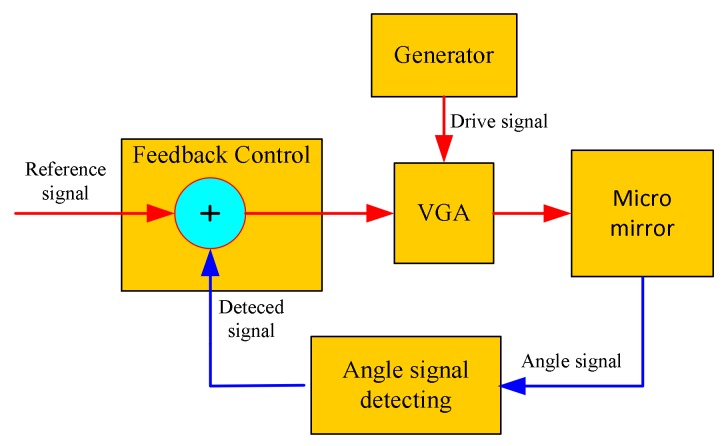
Closed-loop feedback control system.

As the core device of the micro-NIR spectrometer, the MOEMS scanning grating mirror must meet the requirements that are equal to the amplitude of the swing. However, in the working process, the amplitude of the swing of the MOEMS scanning grating mirror will change because of the thermal effects, the environmental factor and other factors, so the closed-loop feedback control must be added to ensure the amplitude of the swing of the scanning grating mirror constantly. Figure 3 shows the closed-loop feedback control system of the scanning grating mirror. The deviation signal is obtained by comparing the reference signal with the obtained detection signal, and the deviation signal can control the drive signal through the variable gain amplifier (VGA), and the amplitude of the swing of the scanning grating mirror is constant. The closed-loop feedback control system can suppress or eliminate the deviation to make the scanning grating mirror swing constantly.

**Figure 4:**
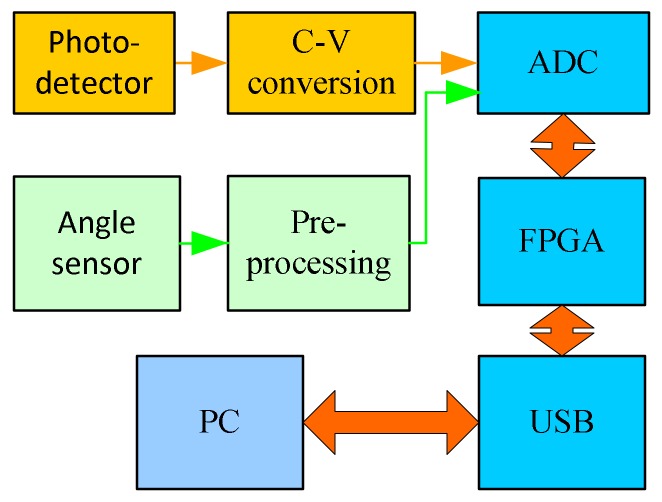
Structure of the signal detecting system.

Figure 4 shows the structure of the signal detecting system of the micro-NIR spectrometer based on the MOEMS scanning grating micrometer, which mainly includes a current voltage (C-V) conversion module, a signal preprocessing module, an analog-digital conversion (ADC) module, an fields programmable gate array(FPGA) control module, and a USB interface module. The FPGA module receives the command from the host computer and controls the spectrometer. The InGaAs single photodetector diode converts the spectrum signal into an electrical signal and transmits it to the ADC module. At the same time, the output angle signal of the angle sensor is also transmitted to the ADC module circuit after pre-processing and the ADC module converts all these analog signals into digital signals. The output signal of the ADC was transmitted into the FPGA module, and to the host computer through a USB interface after FPGA processing, the computer destroys the spectrum information.

## 3. The Circuits and System Design

According to the principles of the control and detection system of the micro-NIR spectrometer that are shown in Figure 3 and Figure 4, the mirror control circuit and the spectrum and angle signal detecting circuits are designed and manufactured.

### 3.1. The Mirror Control System

The previous study found that there is a good linear relationship between the scanning grating mirror drive signal and the output signal of the angle sensor [26]. When the drive signal is a sinusoidal drive signal, the angular sensor output is also a sinusoidal signal.

#### 3.1.1. The Drive Signal Generate Module

**Figure 5:**
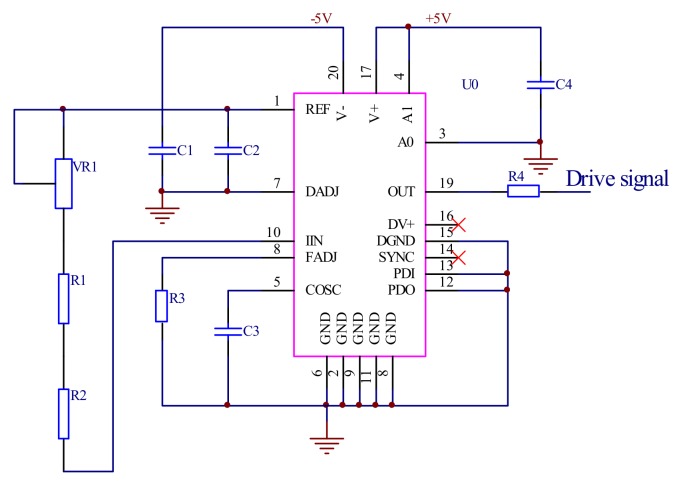
The drive signal generating circuit.

The MAX038 was used to produce a sinusoidal drive signal, as shown in Figure 5. MAX038 is a high frequency precision function signal generator integrated circuit of the Maxim Integrated (San Jose, CA, USA), the frequency of the drive signal is calculated by Equation (1):

(1) $$F(\mathrm{kHz})=2V_{REF}÷[R_{IN}\times C_{F}(nF)]$$

where *V_REF_* is a constant voltage equal to 2.5 V which independent of the power supply, and *R_IN_* is the resistor between *REF* and *IIN* which includes *VR*1, *R*1, and *R*2. *C_F_* is the capacitance connected to *COSC* and *GND* which includes *C*3. The resistance *R*1 is a thermal resistor and its temperature characteristic is opposite to *VR*1 and *R*2, which can effectively eliminate the change in oscillation frequency with the change in temperature.

#### 3.1.2. The Amplitude Detection Module

The feedback signal is generate by comparing the output signal of the angle sensor with the reference signal in the feedback control module. The reference signal is the direct current (DC) and the output signal of the angle sensor is the alternating current (AC). The AC signal output by the angle sensor must be converted into a DC signal. The multiplier AD633 of the Analog Devices (Norwood, MA, USA) is used to convert the AC signal into a DC signal to realize the amplitude of the output angle signal detection. When there are two input signals *U_s_* and *U_r_*, the output signal *U_o_* of the amplitude detection circuit can be calculated by Equations (2)–(4).

(2) $$U_{s}=E_{s}\sin(\omega t+\phi_{1})$$

(3) $$U_{r}=E_{r}\sin(\omega t+\phi_{2})$$

(4) $$U_{o}=U_{s}U_{r}=\frac{E_{s}E_{r}}{2}\left\{\cos\left(\phi_{1}-\phi_{2}\right)-\cos\left[2\omega t+(\phi_{1}-\phi_{2})\right]\right\}$$

where *U_s_* is the angle sensor signal, *U_r_* is the control signal generated by the oscillating circuit, *E_s_* is the amplitude of *U_s_*, *E_r_* is the amplitude of *U_r_*, *ω* is the frequency of the drive signal and the angle sensor signal, *Φ*_1_ is the phase of the angle sensor signal, and *Φ*_2_ is the phase of the reference signal.

**Figure 6:**
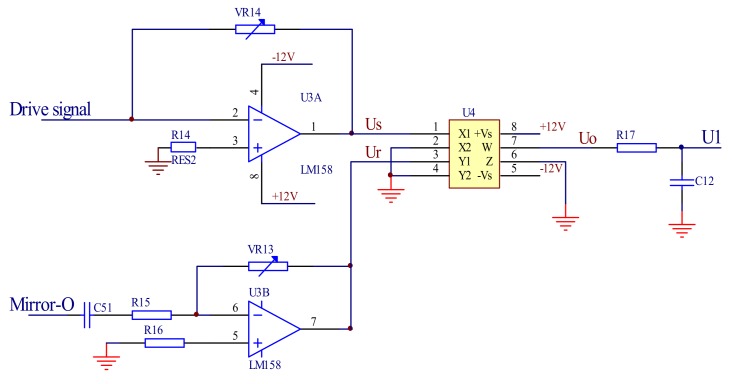
The amplitude detection circuit.

The output signal *U_o_* includes a DC voltage and AC signal and *E_s_E_r_*(cos(*Φ*_1_ − *Φ*_2_)/2 is the DC part. *E_s_E_r_*{[cos(2*ωt* + (*Φ*_1_ − *Φ*_2_)]}/2 is the AC part, which has a frequency that is twice as large as the drive signal, so that it can be removed by the followed low-pass filter and the remained DC part is proportional to the amplitude of the output signal of the angle sensor. Figure 6 shows the schematic of the amplitude detection circuit.

#### 3.1.3. The Feedback Control Module

**Figure 7:**
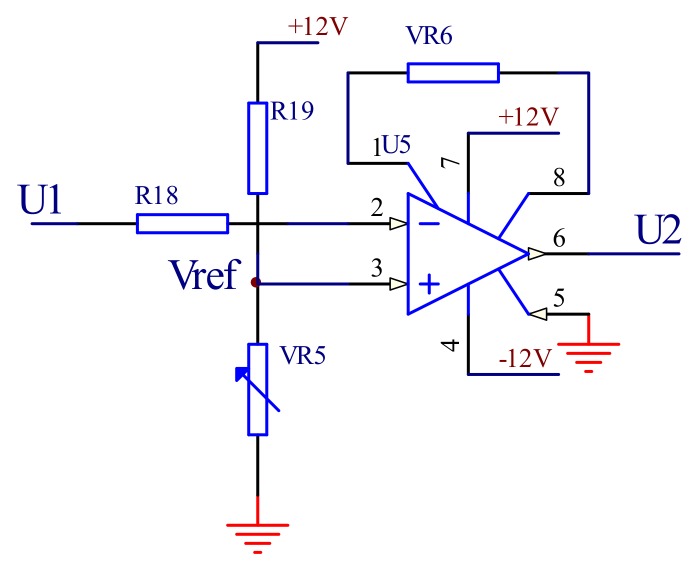
The feedback control circuit.

The feedback control module is composed of a differential comparison circuit. The main function of this module is to compare the output voltage of the amplitude detection module with the reference voltage and to output the deviation voltage. As shown in Figure 7, the reference voltage is connected to the non-inverting input of the amplifier AD620 of the Analog Devices (Norwood, MA, USA), and the reference voltage is set by adjusting the sliding resistor *VR*5. The inverting input terminal is connected with the output of the amplitude detection circuit, and the output signal of the circuit *U*_2_ can be expressed as

(5) $$U_{2}=K\times (V_{ref}-U_{1})$$

where *V_ref_* is the differential signal, the value of the mirror for the balance of peak DC electrical output value of the circuit, and *Vo* for the amplitude detection circuit output signal.

#### 3.1.4. The Variable Gain Amplification Module

**Figure 8:**
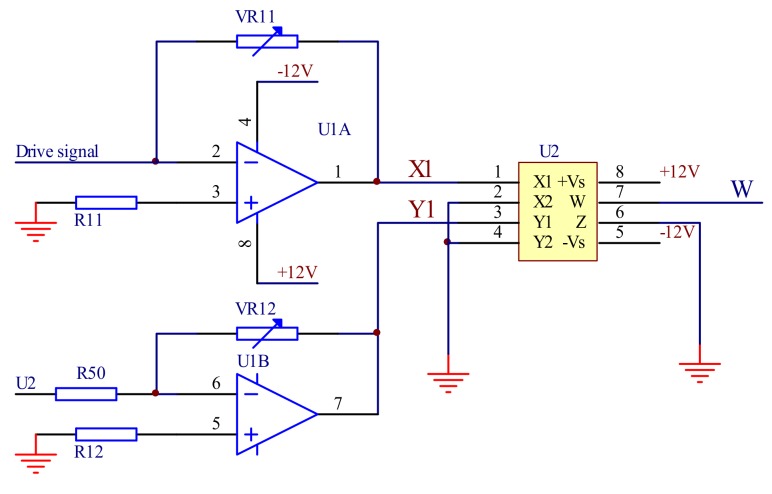
The variable gain amplification circuit.

The four-quadrant multiplier AD633 is used to build the variable gain amplification module. The output signal of the feedback control module is multiplied with the sinusoidal signal generated by the signal generating module. The output signal is used as the MOEMS scanning control system for the MOEMS scanning precise control of the grating mirror. Figure 8 shows the schematic of the variable gain amplification circuit

The output of the feedback control module is connected to *x*; the sinusoidal signal of the drive signal module generated is connected to *y*1 and *x*2, *y*2, and *z* are connected to the ground. The circuit output signal *W* of the module is expressed as Equation (6)

(6) $$W=\frac{x1\times x2}{10}$$

where *W* is the output of the variable gain amplification circuit, which controls the swing angle of the mirror, *x*1 is the drive signal of the mirror that has been amplified, and *x*2 is the output of the feedback control circuit.

Since the amplitude and frequency of the sinusoidal signal generated by the drive signal module have been determined, the output is determined only by the output of the feedback control module; that is, the output of the controllable gain amplification module is controlled by the output signal of the feedback control module.

### 3.2. The Signal Detecting System

The detecting circuit of the detector is working in the photovoltaic mode. In this mode, the InGaAs single photodetector diode hamamatsu G12181-005K (Tokyo, Japan) does not need voltage and there is no dark current, so the output signal can be measured more accurately and the linearity of the output signal is better. The USB interface circuit is designed based on the CYPRESS 68013 series chip (Cypress Semiconductor, San Jose, CA, USA). The FPGA control circuit is designed based on the Altera Cyclone EP2C series chip (San Jose, CA, USA).

#### 3.2.1. The Field Programmable Gate Array (FPGA) Main Control Module

**Figure 9:**
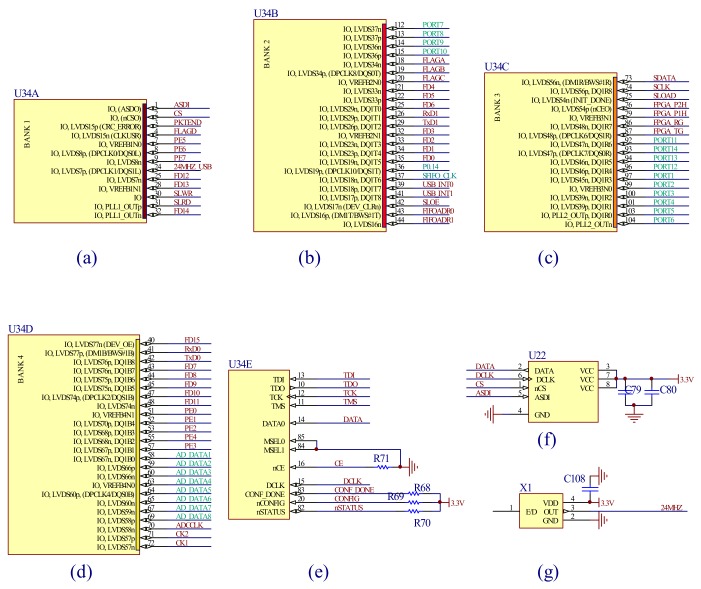
The field programmable gate array (FPGA) main control circuit: (**a**) BANK 1, (**b**) BANK 2, (**c**) BANK 3 and (**d**) BANK 4 of the FPGA; (**e**) the program download interface; (**f**) the memory chip, (**g**) the crystal oscillator circuit.

The FPGA is the core of the detecting circuit, which realizes the sequence of the control and detecting system. The control of the mirror and the module of the ADC and the USB interface need to work synchronously. Furthermore, according to the design requirements of the spectrometer resolution, the resonant frequency of the mirror is 650.30 Hz, and the swing is about 1.5 ms. Thus, the detecting circuit needs to sample at least 4096 points of both the spectral signal and the angle signals simultaneously during the period of a half-cycle (0.75 ms). Thus, the FPGA must working at a high speed and requires a huge data buffer space. Figure 9 shows the FPGA circuit designed with the Altera’s Cyclone II FPGA chip EP2C5T144C8N of the Altera Corporation (San Jose, CA, USA). The chip was divided into four main areas BANK1–BANK4 (a–d), (e) is the program module of the FPGA, (f) is the crystal oscillator circuit which provides the clock of the FPGA and USB, and (g) is the memory chip EPCS4SI8 of the Altera Corporation (San Jose, CA, USA).

#### 3.2.2. The Synchronous Trigger Module

**Figure 10:**
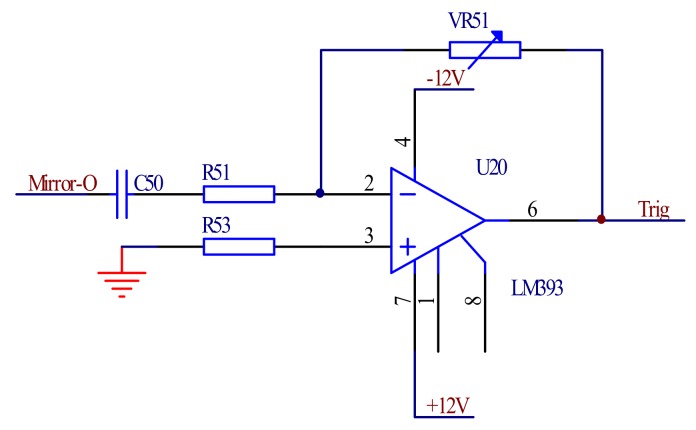
The synchronous trigger circuit.

The output of the angle sensor reflects the state of the mirror swinging angle and determines the starting point of the mirror and the work period of the spectrum signal. The output of the angle sensor is used as the synchronization trigger signal of the signal detecting circuit, so that the comparators can use it to realize the synchronization of the angle signal and the spectrum signal. The output signal of the angle sensor is amplified and shaped, and the output regular square wave of the comparator is used as the synchrony pulse of the signal detecting circuit. Figure 10 shows the schematic of the synchronous trigger circuit.

#### 3.2.3. The Analog-Digital Conversion (ADC) Module

**Figure 11:**
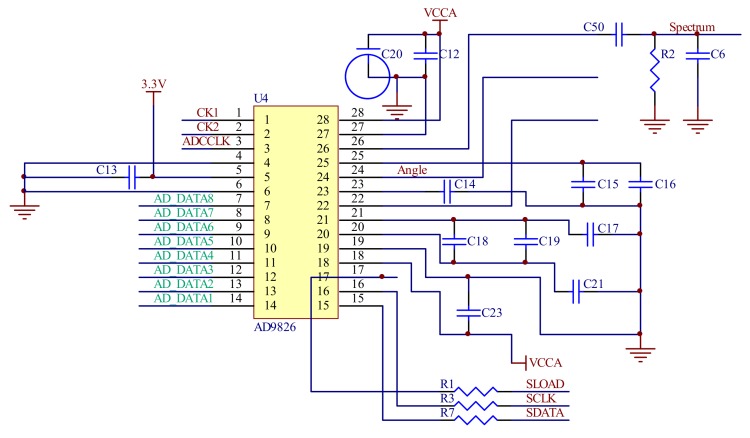
The analog-digital conversion (ADC) circuit of the signal detecting system.

In order to obtain a higher precision swing amplitude of the mirror and a higher acquisition rate of the spectrum signal, the ADC chip must have sufficient sample bits and a sufficient sampling rate. Figure 11 shows the ADC circuit of the signal detecting module. The AD9826 of the Analog Devices (Norwood, MA, USA) has three sampling channels, 16 bit sample accuracy, and a sampling rate of up to 15 Mbps, which meet the requirements of the spectrum and signal sampling.

#### 3.2.4. The Universal Serial Bus (USB) Interface Module

**Figure 12:**
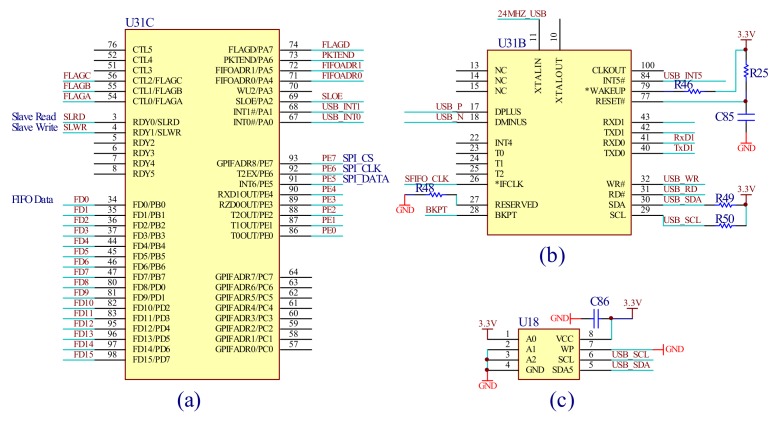
The universal serial bus(USB) interface circuit: (**a**) the main In/Out port of the USB; (**b**) the functional module of the USB and (**c**) the memory chip of the USB for program storage.

The collected spectrum data and angle signals are transmitted to the computer to be reconstructed. Due to the huge amount of information, the USB interface can realize high-speed data transmission. The designed USB2.0 mode can transmit data up to 480 Mbps rate. Figure 12 shows the USB interface circuit, the interface chip is CY7C68013 of the Cypress Semiconductor (San Jose, CA, USA), (a) provides the main In/Out port of the USB interface circuit, (b) is the functional module of the USB and (c) is the memory chip of the USB for program storage.

The host computer receives the spectrum and angle data through the USB interface and reconstructs the spectrum according to the mathematical relationship between the wavelength λ and the micromanipulation angle β.

## 4. Experiment and Result

**Figure 13:**
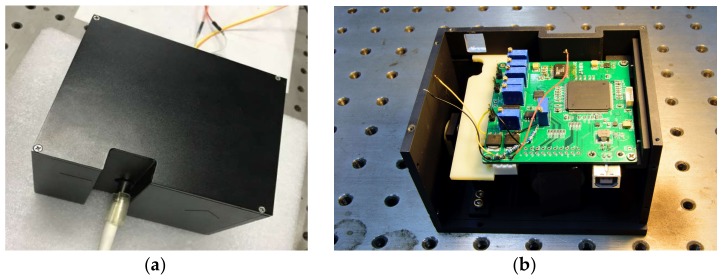
Photographs of the assembled micro-NIR spectrometer: (**a**) external form and (**b**) the inner printed circuit board (PCB).

Figure 13 shows the micro-NIR spectrometer based on the MOEMS scanning grating mirror assembled with the system circuit PCB, the length and width and height of the micro-NIR spectrometer is 104 mm × 84 mm × 74 mm.

The experimental system was set up to test the performance of the circuit and spectrometer. Firstly, the resonant frequency and tilt angle of the grating mirror were measured by laser projection method. The resonant frequency is 650.30 Hz; The tilt angle of the grating mirror by applied different drive voltage is shown it the Table 1, the mirror scanning with the drive voltage varying from 1000 mV to 1000 V, where the maximal scanning angle reaches 9.26° at 1000 mV, and the linear correlation coefficient of the fitting curve of the least square method reaches 0.9999. And the maximum deviation angle is 0.05° at the tilt angle equal to 8.475° between 100 mV and 1000 mV, so the precision of the closed loop control reaches 0.06%. 

**Figure 14:**
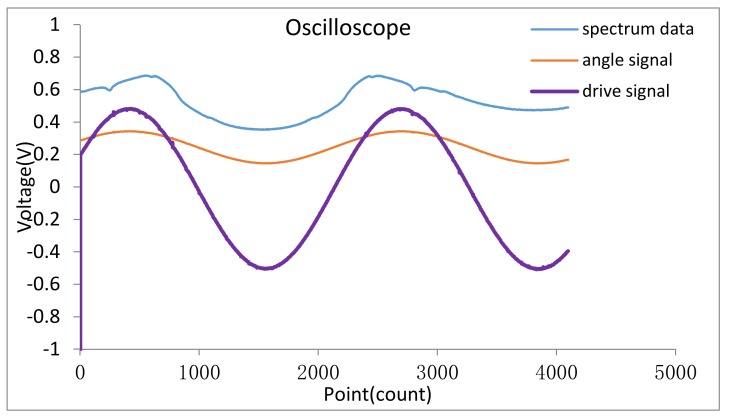
The original spectrum curve and angle signal curve.

Next, the bandwidth of the closed loop control system is measured about 500 KHz frequency, which far beyond the working frequency of the mirror. When the drive voltage with frequency 656.30 Hz and amplitude 500 mV, the tilt angle of the mirror is 5.02° which be able to cover all the wavelength from 800 nm to 1800 nm. The drive voltage and the output signal of the angle sensor and the original spectral data were sampled with an oscilloscope, and the datum was plotted in the Origin software (Origin 9.1, OriginLab Corporation, Northampton, MA, USA) as shown in Figure 14, the frequency of the drive is 650.30 Hz and the amplitude is 500 mV, the frequency of the output voltage signal of the angle sensor is the same as the drive signal and the amplitude is 99 mV. At the same time, the signal-to-noise ratio (SNR) of the spectrometer was tested and found to be 4562 at full scale (65535 code), the spectrum data drift is 0.9% in 24 h.

**Figure 15:**
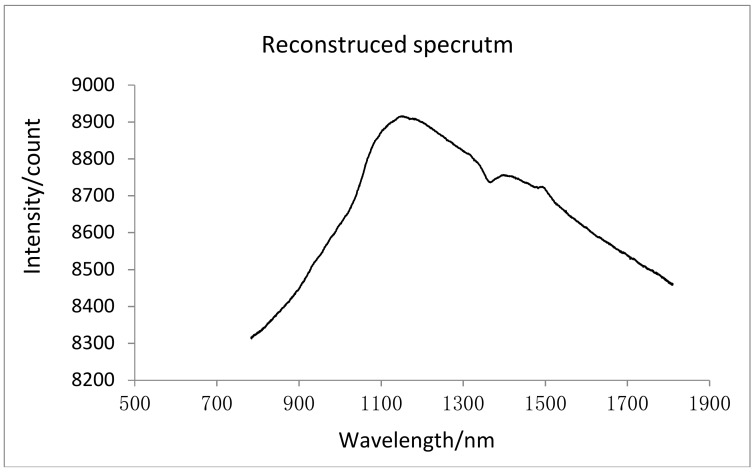
The reconstructed spectrum curve.

Furthermore, the reconstructed spectrum drawn in the Origin software according to the relationship between the original spectrum and the angle single, that are shown in Figure 15.

**Figure 16:**
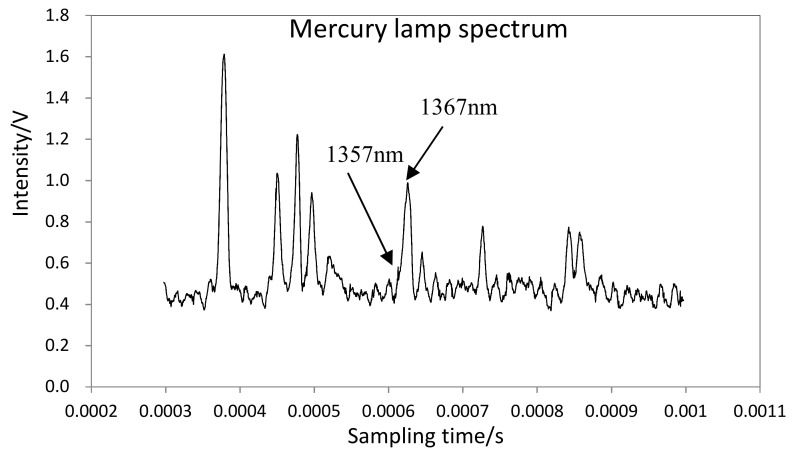
The resolution test curve of the micro-NIR spectrometer.

Finally, the resolution of the spectrometer was tested with a mercury lamp. Figure 16 shows the result of this test. The two adjacent peaks of 1357 nm and 1367 nm can be detected, so the result shows that the resolution of the spectrometer reaches 10 nm in the wavelength range of 800–1800 nm.

## 5. Conclusions

The results show that the control and detecting system of the micro-NIR spectrometer based on a MOEMS scanning grating mirror realizes the stable swing of the mirror, and realizes the effective signal detection of the spectrum signal and correct data transmission. The precision of the closed loop control is 0.06%, and the spectrum data drift is 0.9% in 24 hours; the signal-to-noise ratio (SNR) of the spectrometer reaches 4562 at full scale.

## Figures and Tables

**Table 1:** Measured tilt angle and deviation angle versus applied drive voltage.

Drive Voltage (mV)	100	200	300	400	500	600	700	800	900	1000
Tilt Angle (°)	1.522	2.393	3.220	4.097	5.002	5.865	6.735	7.611	8.475	9.260
Deviation Angle (°)	0.027	0.025	0.027	0.040	0.035	0.040	0.045	0.042	0.050	0.048
